# Advances in the application of functional nanomaterial and cold plasma for the fresh‐keeping active packaging of meat

**DOI:** 10.1002/fsn3.3540

**Published:** 2023-07-09

**Authors:** Muhammad Umair, Tayyaba Sultana, Song Xun, Saqib Jabbar, Muhammad Shahid Riaz Rajoka, Amgad Albahi, Muhammad Abid, Muhammad Modassar Ali Nawaz Ranjha, Hesham R. El‐Seedi, Fengwei Xie, Kashif ur Rehman Khan, Zhao Liqing, He Zhendan

**Affiliations:** ^1^ College of Pharmacy Shenzhen Technology University Shenzhen China; ^2^ Department of Food Science and Technology, College of Chemistry and Environmental Engineering Shenzhen University Shenzhen China; ^3^ College of Public Administration Nanjing Agriculture University Nanjing China; ^4^ National Agricultural Research Centre (NARC) Food Science Research Institute (FSRI) Islamabad Pakistan; ^5^ National Food Research Centre, Khartoum Ministry of Agriculture and Natural Resources Khartoum Sudan; ^6^ Institute of Food and Nutritional Sciences, Pir Mehr Ali Shah, Arid Agriculture University Rawalpindi Pakistan; ^7^ Institute of Food Science and Nutrition, University of Sargodha Sargodha Pakistan; ^8^ Department of Chemistry, Faculty of Science Islamic University of Madinah Madinah Al Madinah Al Munawwarah Saudi Arabia; ^9^ International Research Center for Food Nutrition and Safety Jiangsu University Zhenjiang China; ^10^ School of Engineering Newcastle University Newcastle upon Tyne UK; ^11^ Department of Pharmaceutical Chemistry, Faculty of Pharmacy The Islamia University of Bahawalpur Bahawalpur Pakistan

**Keywords:** active packaging, bacteriostasis, nanomaterial, nonthermal, photocatalytic, sterilization

## Abstract

The most recent advancements in food science and technology include cold sterilization of food and fresh‐keeping packaging. Active packaging technology has received much interest due to the photocatalytic activity (PCA) of functional nanoparticles, including titanium dioxide (TiO_2_) and ferric oxide (Fe_2_O_3_). However, there are still significant concerns about the toxicity and safety of these functional nanoparticles. This review emphasizes the bacteriostatic and fresh‐keeping properties of functional nanoparticles as well as their packaging strategies using the ultraviolet photo‐catalysis effect. High‐voltage electric field cold plasma (HVEF‐CP) is the most innovative method of cold‐sterilizing food. HVEF‐CP sterilizes by producing photoelectrons, ions, and active free radicals on food media, which come into contact with the bacteria's surface and destroy their cells. Next, this review also assesses the photocatalytic activity and bacteriostasis kinetics of nanosized TiO_2_ and Fe_2_O_3_ in poultry, beef, and lamb. In addition, this review also emphasizes the importance of exploiting the complex interaction processes between TiO_2_ and Fe_2_O_3_, along with dietary components and their utilization in the fresh meat industry.

## Graphical abstract

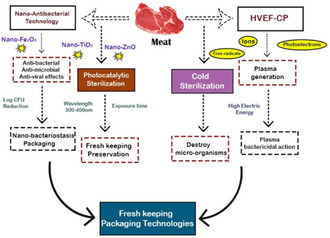

## 1 INTRODUCTION

In the last few decades, many industries, including the food industry, have started to use polymeric materials instead of traditional food packaging materials such as glass, metal, and paper. This might be due to the substantial cost savings and physical and chemical similarities between polymers and conventional materials. Polymeric packaging materials are also more flexible, transparent, lightweight, and chemical resistant. On the other hand, polymer surfaces usually exhibit low free surface energy and are both hydrophobic and hydrophilic in nature Qian et al., 2019. Polymers, therefore, lack the distinctive surface characteristics needed for various applications. Additionally, it is expensive to produce multilayer, structured food packaging polymers. To create polymers with the desired properties, several surface treatments are applied.

Surface treatments for packaging materials can serve many purposes, such as surface functionalization, cleaning or etching the surface, and adding something to the surface. Surface functionalization is the process of adding certain functional groups to the surface layer of a polymer. Surface functionalization of polymers is often used to improve wetting, sealing, printing, dye absorption, resistance to glazing, or adhesion to other polymers or materials without changing the bulk properties of the polymer (Hah et al., 2021; Tyuftin & Kerry, 2020). In addition, surface functionalization was utilized to improve the efficacy of polymer food packaging barriers and impart antibacterial properties (Wong et al., 2020). Surface treatments can also be utilized to sanitize or etch polymer surfaces by removing undesirable elements from the surface layers. In addition, surface treatments can be used to sterilize or add thin coatings to the surface of polymers (Hamdi et al., 2020).

Surface modification of polymers is possible via chemical or physical processes. Popularity‐wise, physical methods have transcended chemical procedures due to their superior precision, controllability, and environmental benevolence. Traditional methods for modifying polymer surfaces include flame and corona treatment, ultraviolet, gamma, ion beam techniques, low‐pressure plasma, and laser therapy (Farooq et al., 2020). Due to the relatively short duration of the enhanced properties, flame and corona treatments for polymers are not particularly effective (Lazar et al., 2020).

In contrast, when HVEF‐CP is applied to polymers, the plasma–polymer interface initiates an assortment of chemical and physical reactions that alter the surface properties. This was used to give the packaging polymer specific and controllable surface energies to improve adhesion or sometimes antiadhesion, printability, sealability, antimist properties, and resistance to mechanical failure (Kehrer et al., 2020). Plasma deposition of barrier layers can make packaging materials better at blocking gases (like oxygen and carbon dioxide) and chemical solutions. Additionally, gas plasma reactions can quickly and effectively kill microorganisms (like bacterial cells, spores, yeasts, and fungi) that adhere to polymer surfaces (Umair et al., 2020). Using cold plasma, plastic bottles, caps, and sheets can be sterilized quickly without changing their shape or leaving residue behind (Rutala et al., 2020). Following a brief discussion of the physics and chemistry of cold plasma, this review further describes the most recent applications of cold plasma technology for modifying polymers used in food packaging. Approximately 80% of the polymers used in food packaging are polyethylene, polypropylene, and polyethylene terephthalate (Siracusa & Blanco, 2020). In addition to identifying gaps in knowledge, the review guides future research initiatives.

## 2 PURPOSE AND SIGNIFICANCE OF THE RESEARCH

Food that is vulnerable to heat sources is susceptible to spoilage due to insufficient sterilization, which reduces shelf life and makes long‐distance transportation difficult (Doulgeraki et al., 2012).

The gas composition of modified atmosphere packaging (MAP) significantly impacts the survival and proliferation of spoilage‐causing microorganisms in meat and meat products (Kolbeck et al., 2021; Nychas & Skandamis, 2005). The spoilage rate of meat stored under aerobic conditions is well documented, owing to the rapid propagation of *Pseudomonas* spp. and facultative anaerobes, which are the main microflora in vacuum packaging (VP) and MAP. Moreover, Psychrophilic bacteria and *Clostridium* spp. have also been identified as major contributors to the spoilage of vacuum‐packed and chilled meat (Mills et al., 2014; Anas et al., 2019; Wambui & Stephan, 2019). The dynamics of meat spoilage‐causing bacteria have recently been observed in several studies (Wambui & Stephan, 2019). Nonetheless, the effects of packaging on a specific species or strain of a specific genus have been limited Abdelgader, et al., 2018. Furthermore, previous studies have shown that bacterial growth in fresh and chilled meat is affected by storage time and packaging conditions (Ercolini, Ferrocino, et al., 2010; Pennacchia et al., 2011).

The most common Psychrophilic bacteria that cause spoilage in meat under aerobic conditions, even at very low storage temperatures, are *Pseudomonas fluorescens*, *Pseudomonas fragi*, *Pseudomonas lundensis*, and *Pseudomonas putida* (Ercolini et al., 2007; Ercolini, Casaburi, et al., 2010). *P*. *fluorescens* strain was more dominant in fresh meat at an earlier stage of storage than *P*. *fragi* strain, but *P*. *fragi* strain became the most prevalent *Pseudomonas* spp. in stored meat at a later stage of storage (de W Blackburn, 2006). Furthermore, *P*. *fragi* was identified as the most common bacteria responsible for spoilage, followed by *P*. *lundensis* and *P*. *fluorescens*. The growth of *P*. *fluorescens* and *P*. *fragi* in red meat can be inhibited at higher CO_2_ concentrations (more than 10%). Furthermore, CO_2_ had a more significant effect on *Pseudomonas fragi* than on *Pseudomonas fluorescens* and *Pseudomonas lundensis* (de W Blackburn, 2006). According to another researcher, *P. fragi* was discovered during PCR analysis in all kept meat samples, including air packing, MAP, and VP (Ercolini et al., 2007, 2011). In addition to the *P. fragi* strain, Olofsson et al. (2007) detected many novel *Pseudomonas* spp. in frozen meat. The presence of *P. fragi* in stored meat samples following air packing, MAP, and VP, on the other hand, suggests that *P. fragi* is one of the most significant *Pseudomonas* spp. implicated in meat deterioration Anas et al., 2019; Shahein et al., 2022.

In addition to MAP and VP, there is another preservative approach using ultraviolet light (Ercolini, Casaburi, et al., 2010), but the ultraviolet preservative effect for fresh and chilled meat is inadequate due to ultraviolet's lower penetration ability into food packaging materials. However, the use of photocatalytic nanoparticles as antibacterial packaging is limited. As a result, increasing ultraviolet penetration in packaging is critical, as it provides fundamental theoretical support for the development of photocatalytic antimicrobial packaging materials and their application in fresh meat packaging technology (Ercolini et al., 2011).

Previous improvements in packaging technology and ways to sterilize fresh meat and meat products are still insufficient. Ercolini, Casaburi, et al. (2010) found that even when food is stored and moved at low temperatures, surface microorganisms can grow rapidly, causing spoilage and short shelf life. Also, changes in temperature during thermal sterilization affect the meat's taste, texture, physical and chemical properties, and nutritional value, making it hard to keep the meat's original quality. The food industry has had to develop fresh‐keeping packaging technology because customers want food to be safe and consistent while still keeping its original quality.

In recent years, nano‐antimicrobial and fresh‐keeping packaging technology has been used in a range of meat packaging, including mutton, tuna, and poultry (Ameta et al., 2020). Also, Chaudhary et al. (2020) found that certain nano‐inorganic materials with photocatalytic and bacteriostatic properties are used in food packaging materials. These free radicals can invade microorganisms, ultimately enhancing the quality of meat and meat products (Nasiru et al., 2021; Fang et al., 2017). Moreover, the functional properties of nanomaterial‐based food packaging materials can be stimulated in the presence of light, which can impede microorganisms on food surface and slow down microbial replication during food fermentation, shipping, and storage, thus extending commodity shelf life (Ameta et al., 2020). At the same time, the heat produced during the illumination phase is comparatively low, so the consistency of the goods is not affected, essentially preserving the food's specific taste quality (Al‐Tayyar et al., 2020). Nano‐antimicrobial and fresh‐keeping packaging technologies have distinct advantages in preserving product quality and extending shelf life, especially in the packaging of heat‐sensitive foods, and these technologies will have a wide range of applications.

Plasma technology is commonly employed in material production, and it represents a significant technical advancement in the processing of food and agricultural products (Umair et al., 2020). The DBD system's cold source plasma is one of the most popular plasma technologies, known as sealed packed plasma technology of ions in packages (Umair et al., 2021). The DBD plasma device will accommodate the entire package without the need for electrodes inside. It is convenient to use. The benefit of using plasma sterilization is that, after packaging, it would not cause secondary contamination. The sterilizing agent is derived from gas, leaves no chemical traces, and is nontoxic and harmless. Plasma technology from cold sources can generate highly efficient low‐voltage bactericidal agents that can efficiently sterilize the product.

There have been few reports on the bactericidal impact of food packaging. Fresh meat cannot be sterilized with a heat source (Wołoszyn et al., 2020). It is critical to study and improve cold sterilization methods for such foods, as they have significant potential for industrial use. Although the desired voltage in the plasma generation process is very high, it does not emit excessive heat, which prevents a dramatic rise in the treatment temperature (Umair et al., 2021). As a result, plasma sterilization technology can be used to sterilize fresh meat and heat‐sensitive foods using a novel cold purification process. Furthermore, plasma encompasses diverse active substances that can kill food spoilage‐causing microorganisms, and cold plasma treatment may emit ultraviolet light, which may damage microorganisms (Umair et al., 2020). Ultraviolet, on the other hand, can stimulate the activity of functional nanomaterials. However, the penetration ability of ultraviolet into packaging material can also affect the efficiency of the process (Wang et al., 2020). Consequently, incorporating plasma technology into functional nanoparticles can synergistically boost the action of nanomaterials, resulting in stronger inhibition of microbes on the surface of packed meat products and extended shelf life.

## 3 SPOILAGE MICROORGANISMS IN MEAT AND MEAT PRODUCTS

Storage temperature has an important effect on spoilage bacteria growth and chicken spoilage in tuna meat (Nakazawa et al., 2020). When fresh chicken is kept at low temperatures, most microorganisms cannot grow or reproduce well in this environment. When considering the relationship between temperature and microorganisms, two points must be considered: the temperature of the microorganisms and the time in which they exist. Psychrophilic bacteria can grow at freezing temperatures and cause food spoilage (Hur et al., 2009). However, their growth and reproduction rates are significantly reduced at low temperatures (Nakazawa & Okazaki, 2020). Most thermophilic bacteria cannot grow and propagate at 5°C. At low temperatures, the growth and reproduction of *Escherichia coli* are obviously restricted, which shows not only the prolongation of the reproductive cycle but also the logarithmic phase.

Controlling chicken spoilage and prolonging shelf life is important for chicken and chicken products. The leading causes of chicken spoilage are long‐term storage, inappropriate storage temperature, high concentrations of contaminated microorganisms, and high pH after stiffness. According to Nakazawa and Okazaki (2020), Psychrophilic bacteria growth during storage under frozen aerobic conditions is the main cause of chicken meat spoilage. The principal spoilage bacteria are *Pseudomonas* spp., which emit a typical spoilage odor when their concentration reaches 108 cm^2^. Studies have shown that the microorganisms that appear on the surface of chicken meat immediately after slaughter are usually not the dominant bacteria causing corruption. Although the content of spoilage bacteria in slaughtered chicken is very low, they can grow and reproduce rapidly during storage.

## 4 FRESH MEAT PACKAGING TECHNOLOGY

### 4.1 Vacuum and heat‐shrinkable packaging technology

Currently, vacuum packaging is the most common packaging method in the meat packing market. By pumping most of the air out of the bag and lowering the amount of oxygen in it, vacuum packaging stops myoglobin from oxidizing and keeps its purple color. Ercolini et al. (2007) found that this packaging can create a low‐oxygen environment, slow the growth of microorganisms, stop lipid oxidation, stop microbial contamination from the outside, reduce the meat's moisture content, and keep its appearance.

Vacuum packaging of meat under hypoxic conditions can extend its shelf life by inhibiting the growth and reproduction of microorganisms. However, the material for vacuum packaging has poor air permeability, which has some shortcomings in maintaining the color of meat and meat products and inhibiting the growth of anaerobic microorganisms on the surface of products. A researcher believed that the inhibiting effect of vacuum packaging on microorganisms was realized by the accumulation of CO_2_ in packaging bags. The products used residual oxygen in packaging to breathe and eliminate CO_2_. The accumulation of CO_2_ and the consumption of residual O_2_ in packaging inhibit the growth of aerobic bacteria without affecting lactic acid bacteria (Ercolini, Ferrocino, et al., 2010).

Lactic acid bacteria produce lactic acid and hydrogen peroxide during the growth process. These components can inhibit the growth of other microorganisms. Lactic acid bacteria decompose protein slowly and prolong the storage period of meat to a certain extent. However, the use of vacuum packaging is limited due to the loss of moisture in meat due to the decrease in internal pressure during vacuum heat shrinkage. A better method for fresh‐keeping packaging is to vacuum the product first and then use hot water or hot air treatment so that the packaging bag's heat shrinkage is close to the meat noodles (Jiang et al., 2011). Currently, the vacuum packaging of fresh meat is the form of packaging used in Western countries. Among them, more than 90% of beef in the United States has been packaged by vacuum shrinkage packaging, while heat shrinkage packaging has not been widely used in China.

### 4.2 MAP‐controlled atmosphere fresh‐keeping technology

Modified atmosphere packaging uses the required gas to replace the air in the package. Adjusting the gas environment around meat and meat products can inhibit the growth of microorganisms and maintain the color of the products. The gases commonly used in modified‐atmosphere meat packaging are as follows: (Indicators) Oxygen (O_2_): using a high oxygen composition ratio to produce a higher partial pressure of oxygen, inhibit the growth of anaerobic microorganisms, and form a large amount of oxymyoglobin to maintain the color of muscle. (2) Nitrogen (N_2_): Nitrogen is usually used as an aerator to maintain the balance of internal and external pressure in packaging. Nitrogen has no effect on the color of meat in packaging and has no obvious inhibitory effect on bacteria. (Indicators) Carbon dioxide (CO_2_): Carbon dioxide can change the permeability of bacterial cell walls and affect their pH value and endogenous enzyme activity, thus inhibiting bacterial growth and achieving the goal of prolonging shelf life. It is widely used in MAP (Li et al., 2006).

**FIGURE 1:**
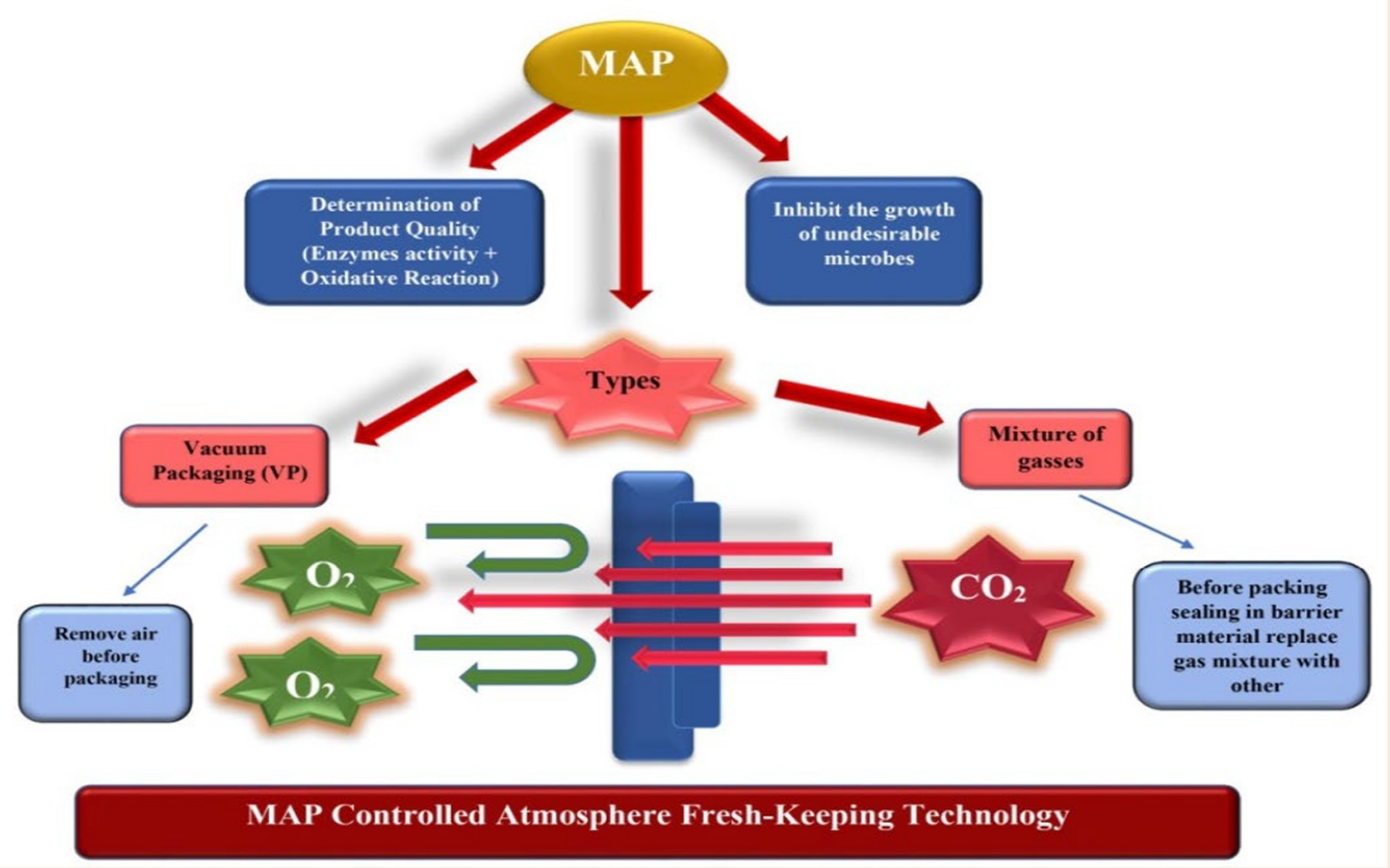
Modified atmosphere packaging (MAP) and its action mode.

The CO_2_ concentration of 30% in MAP had a pronounced antibacterial effect on conditioned beef. Fresh minced beef was packaged in a high‐oxygen atmosphere. The storage period of minced beef was 9 days and 3–4 days at 4.3–8.1°C, respectively, while it could only be stored for 2 days at 15.5°C (Seydim et al., 2006). Fresh ostrich meat is packaged in high oxygen, high nitrogen, vacuum, and air, respectively. The results show that oxygen is the most important factor affecting its shelf life at about 4°C. A research group (Murcia et al., 2003) studied the ingredient stability and shelf life of ready‐to‐eat foods packaged in a vacuum and modified atmosphere. It was found that the shelf life of products could reach 29 days while the composition of food remained unchanged. It is generally believed that the ratio of CO_2_ to 30% of mixed gas will have a significant bacteriostatic effect (Phillips, 2003) MAP and its action mode are illustrated in Figure 1.

### 4.3 Active packaging and antimicrobial packaging and their development trend

Active packaging can not only wrap meat but also have some beneficial effects. Active packaging refers to the interaction between gases and packaging materials in food packaging, which can prolong the shelf life of products, maintain product quality, and improve food safety. Typical main functions of active packaging are deoxidation, diethylene, CO_2_ removal or release, moisture regulation, antimicrobial, odor adsorption, and ethanol release (Chung et al., 2009).

Antimicrobial packaging often combines antimicrobial chemicals with packaging materials and extrudes them with one or more polymers to generate antimicrobial films. The antibacterial agent can be released from the new packaging material and make contact with the bacteria on the food surface (Li et al., 2006).

**FIGURE 2:**
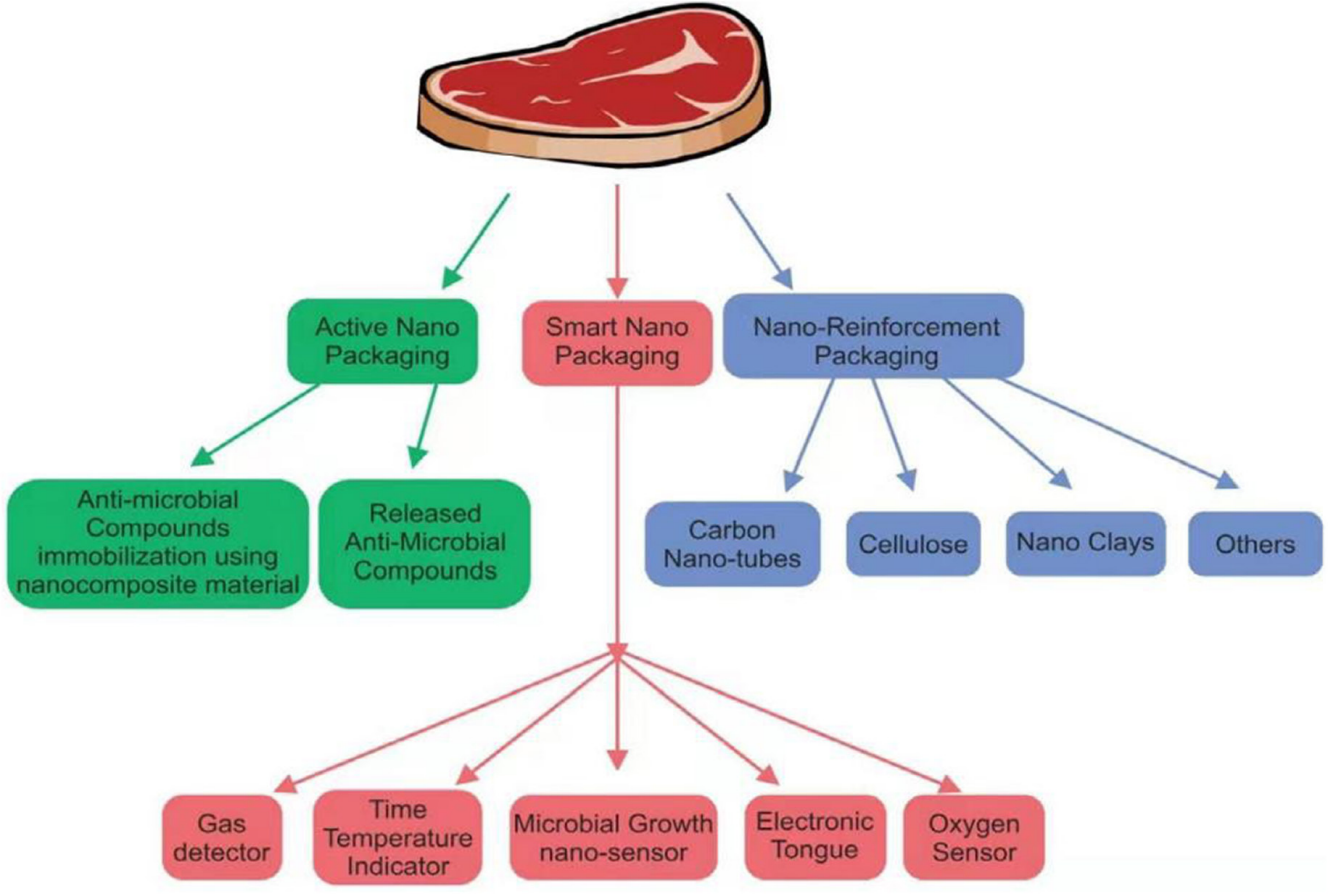
Nano‐antimicrobial and fresh‐keeping packaging technology.

Microorganisms remain on the surface of food during processing, storage, transportation, and treatment. Antimicrobial packaging can kill or inhibit microorganism growth, prolong the shelf life of food, and improve product safety (Chawengkijwanich & Hayata, 2008). Nano‐antimicrobial and fresh‐keeping packaging technology are illustrated in Figure 2.

Fresh meat is a complex food. To prolong the shelf life of meat, the purpose of preservation and packaging should be considered. It is difficult to achieve a single preservation method. Therefore, the combination of various technologies is the trend and direction of meat packaging. As new packaging technologies, active packaging and antimicrobial packaging are flexible in packaging treatment (Chawengkijwanich & Hayata, 2008). Nano‐antimicrobial packaging material can be prepared by combining a polymer matrix with substances with antimicrobial activity (Gondal et al., 2004). Natural antimicrobial extracts, such as tea polyphenols, nutmeg volatile oil, cinnamon volatile oil, nisin, trehalose, mannan, chitosan, etc., have been applied to the antimicrobial preservation of meat (Bandara et al., 2001). The antimicrobial properties of nanomaterials have gradually become a research hotspot in antimicrobial packaging. Nanomaterials with photocatalytic activity are easy to stimulate, do not need specific equipment, and are easy to operate, especially for heat‐sensitive products, which have a far‐reaching impact on maintaining product quality and extending shelf life (Bang et al., 2005).

## 5 NANO‐ANTIBACTERIAL AND FRESH‐KEEPING PACKAGING TECHNOLOGY

### 5.1 Application status of nanotechnology in the food industry

Nanotechnology has been used a lot in food processing, food packaging, food labels, food safety detection, and biosensors. There have been many reports in this research field. However, the main applications of nanotechnology in the packaging industry are nano‐antimicrobial packaging materials, nano‐fresh‐keeping packaging materials, and new high‐barrier packaging materials. Food packaging materials containing nanomaterials will be intellectualized. The development goal is to control food packaging materials according to environmental conditions, self‐repair damages, and warn consumers when food is contaminated or has pathogenic bacteria (Ryan et al., 2002).

In recent years, for food nano‐packaging, polymer‐based nanocomposite has been the most widely studied nanomaterial at home and abroad. Nanomaterials with a molecular level (10 nm) or ultrafine particles are mixed with polymers (such as PA, PE, PP, PVC, etc.), and then new composites are made (Gutierrez et al., 2009). To fulfill the packaging needs of different foods, many new composite materials for food packaging have been introduced, such as nano‐TiO_2_/PP, nano‐Ag/PE, nano‐montmorillonite powder/PA, and so on. The physical, chemical, and biological properties of these new materials have been improved, and their plasticity, stability, barrier, antimicrobial, and fresh‐keeping properties have also been greatly enhanced. New materials have also been applied in the food packaging industry for meat, chicken, and fish, and desired packaging effects have been achieved (Nangmenyi et al., 2011).

Endowing plastic packaging materials with certain antibacterial properties is called nano‐antibacterial packaging material. Films made of 1% silver zeolite added to the masterbatch can completely kill microorganisms causing food poisoning within 1–2 days or coat the surface of containers with this film, which can be widely used in cooked meat, aquatic products, and liquid food packaging (Nangmenyi et al., 2011). Chemical Giant Bayer produces a transparent plastic film containing clay nanoparticles, called Durethan, which prevents fresh meat or other foods from being exposed to oxygen, carbon dioxide, and water. In addition, clay nanoparticles in the film can reduce the quality of the film, enhance its toughness, and improve its heat resistance capacity (Akhavan & Azimirad, 2009).

Compared with traditional nylon plastics, the polyenzymatic amine‐6 plastics (NPA‐6) made by nanocomposite technology have more advantages (Gutierrez et al., 2009). The oxygen and carbon dioxide transmittance of NPA‐6 has been reduced by half, and the water permeability has also been reduced by about 30%. Using NPA‐6 to package meat products such as sausages, ham, and beef jerky can prolong the shelf life of products and maintain product quality (Bandara et al., 2001).

### 5.2 Antibacterial properties and application of nanomaterials

#### 5.2.1 Antibacterial effect of nano‐TiO_2_ and its application

TiO_2_ is an ideal inorganic antibacterial agent with stable chemical properties, is harmless to the human body, and has a low cost. Nano‐titanium dioxide is an important semiconductor metal compound. It usually exists in anatase, rutile, and brookite. The two kinds of nano‐titanium dioxide are the most widely used, and their forms are very stable. The valence band energies of anatase and rutile titania are 3.2 and 3.0 eV, respectively (Trapalis et al., 2003). Anatase is the most active form, and its activity spectrum shows that its activity decreases sharply above 385 nm. A scientist determined the possibility of decomposing cyanide in water with titanium dioxide (Zhang et al., 2008), which proved that it had a promising application in environmental protection.

**FIGURE 3:**
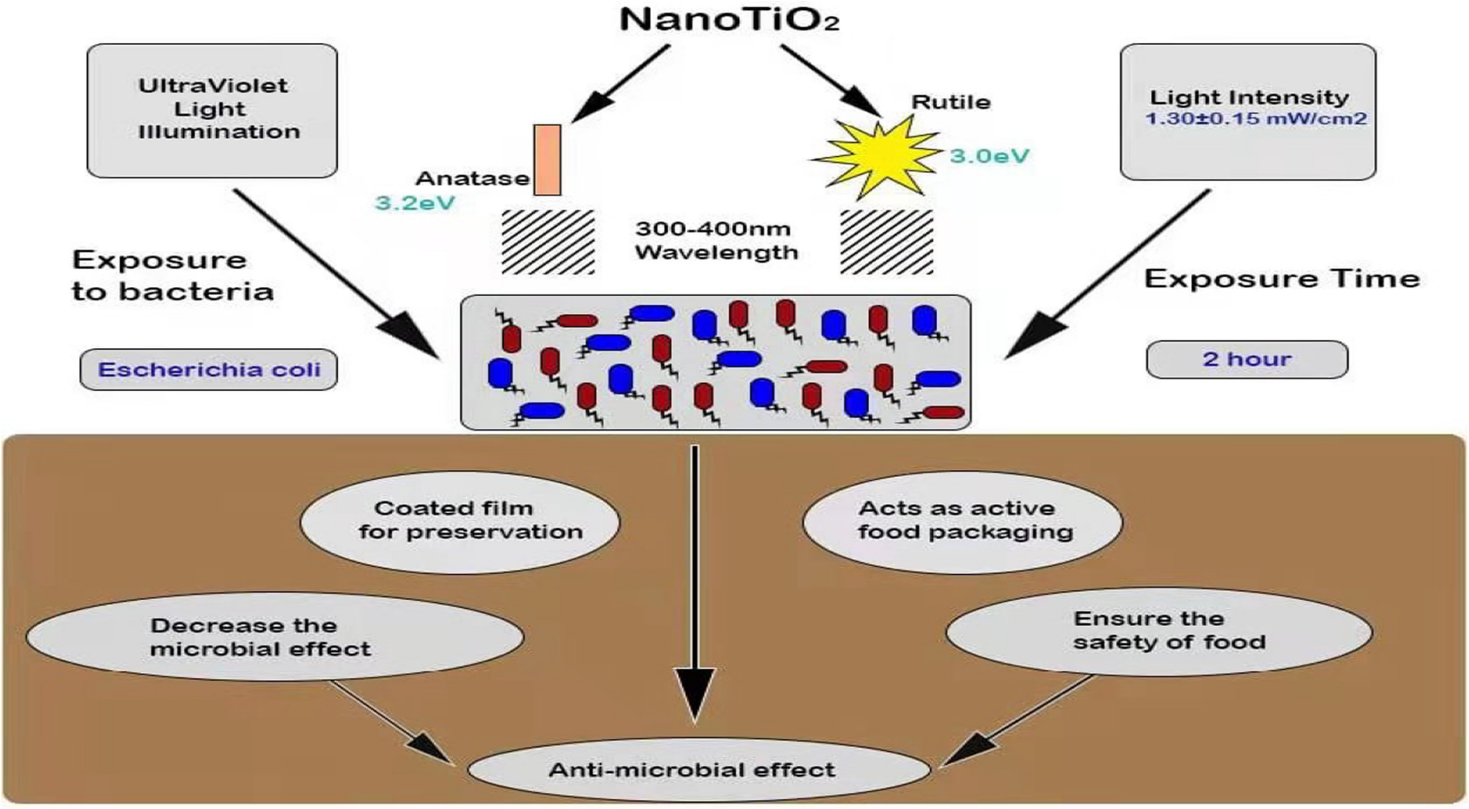
Application of nano‐titanium dioxide on packaging materials.

Nano‐titanium dioxide composite film used in meat packaging can effectively reduce the harmful components such as CO_2_, H_2_, and ethylene produced in the metabolic process, inhibit or kill surface microorganisms, and prevent meat from decaying (Figure 3). The PVC/titanium dioxide nanomaterials were prepared by mixing titanium dioxide nanoparticles with PVC (polyvinyl chloride), which can extend the shelf life (Nangmenyi et al., 2011). Besides its other application, this new nano‐packaging material, made of nano‐powder (titanium dioxide, Ag, and kaolin), was also used to preserve beef in soy sauce. It can effectively inhibit the growth and reproduction of bacteria in soy sauce beef, reduce the production of volatile base nitrogen, prolong the shelf life of soy sauce beef, and maintain the color and flavor of the product (Akhavan & Azimirad, 2009). In green tea preservation, the retention of Vc, chlorophyllic acid, tea polyphenols, and amino acids in the nano‐packaging group was (7.7%, 6.9%, 10.0%, and 2.0%) higher than that in the ordinary packaging group (Murcia et al., 2003).

The application of nano‐titanium dioxide on packaging materials can reduce the degree of microbial contamination on the surface of packaging products and reduce the risk of microbial growth in food. Chawengkijwanich and Hayata (2008) studied the antimicrobial activity of packaging materials coated with titanium dioxide. The results showed that titanium dioxide reduced *E. coli* by 3 CFU/mL after 180 min. The bacteriostasis depends on the intensity of UVA and the type of light source, independent of particle size. The total number of *E. coli* decreased from 6.9 to 4.9 CFU/mL when lettuce was packaged with packing material coated with titanium dioxide and stored under ultraviolet light for 1 day. Table 1 shows the antimicrobial effect of TiO_2_ by applying photocatalytic sterilization.

**TABLE 1:** Antimicrobial effect of TiO_2_ by applying photocatalytic sterilization.

Sterilization technique	Light intensity	Wavelength	Targeted bacteria	Exposure time	Log CFU bacteria reduction	Food application	Antimicrobial effect	References
UV light illumination	1.30 ± 0.15 mW/cm^2^	300–400 nm	*Escherichia coli*	2 h	—	Packaging	Shows photocatalytic activity, maximum transparency, and antimicrobial action	Xie and Hung (2018)
UV light	1 mW/cm^2^	300–400 nm	*E. coli*	180 min	1.5 log CFU/mL	Coated film for preservation	Decrease the growth of microbes and microbial contamination risk	Chawengkijwanich and Hayata (2008)
Fluorescent and UV	—	425 and 365 nm	*E. coli*	3 days	2.23 log CFU/g	Food packaging film	Ensure the safety of food by acting as an antimicrobial agent	Othman et al. (2014)
UV light	300 mW/cm^2^	380 nm	*Pseudomonas fluorescens*	150 min	4 log CFU/mL	Food Preservation	Damages cell walls and cell membranes, which causes cytoplasm leakage and bacterial cell deteriorate	Wang et al. (2014)
UV light	16 W/cm^2^	254 nm	*Salmonella typhimurium*	60 s	6.7 log	Sterilize food packing material	Reduces microbial contamination in fresh carrots	Cho et al. (2007)
Fluorescent light	—	400–500 nm	*E. carotovora*	60 min	—	Packaging thin film	Controls pathogens to reduce the plant diseases	Bodaghi et al. (2013)
UV light	1 μW/cm^2^	300–400 nm	*E. coli*	2 h	2 log CFU/mL	Food packaging	Improves bactericidal activity	Sunada et al. (2003)
UV visible	—	200–800 nm	*P. aeruginosa*	10 min	2 log CFU/mL	Composite Films	Inhibits bacterial growth and retards spoilage of food commodity	Ubonchonlakate et al. (2012)
UV irradiation	—	300–400 nm	*Staphylococcus aureus*	60 min	2.23 log CFU/g	Polyethylene based film	Acts as an active food packaging system to inhibit bacterial growth	Xing et al. (2012)
UV light	500 ± 10 μW/cm^2^	300–400 nm	*Listeria innocua*	30 min	—	Packaging films	Acts as a proficient antibacterial	Bonetta et al. (2013)

#### 5.2.2 Antibacterial effect of nano‐Fe_2_O_3_ and its application

The valence band energy of Fe_2_O_3_ is 2.2 eV, making it a very useful semiconductor and a good candidate material for a photocatalyst. The photocatalytic characteristics of Fe_2_O_3_ have been reported in wastewater treatment (Chawengkijwanich & Hayata, 2008), semiconductor electrode applications (Chawengkijwanich & Hayata, 2008), and photo‐degradation of organic pollutants (Gondal et al., 2004). The results show that nano‐Fe_2_O_3_ can resist aquatic corrosive factors such as viruses, arsenic trioxide, and lead (Bandara et al., 2001; Bang et al., 2005). The surface charge of Fe_2_O_3_ in an aqueous solution originates from the diffusion of hydroxyl groups on the surface, which can effectively remove viruses in water and form surface complexes through electrostatic interaction (Gutierrez et al., 2009). Although nano‐Fe_2_O_3_‐layer fiberglass has been proven to have strong antiviral ability, its antibacterial ability is relatively poor (Ryan et al., 2002).

**FIGURE 4:**
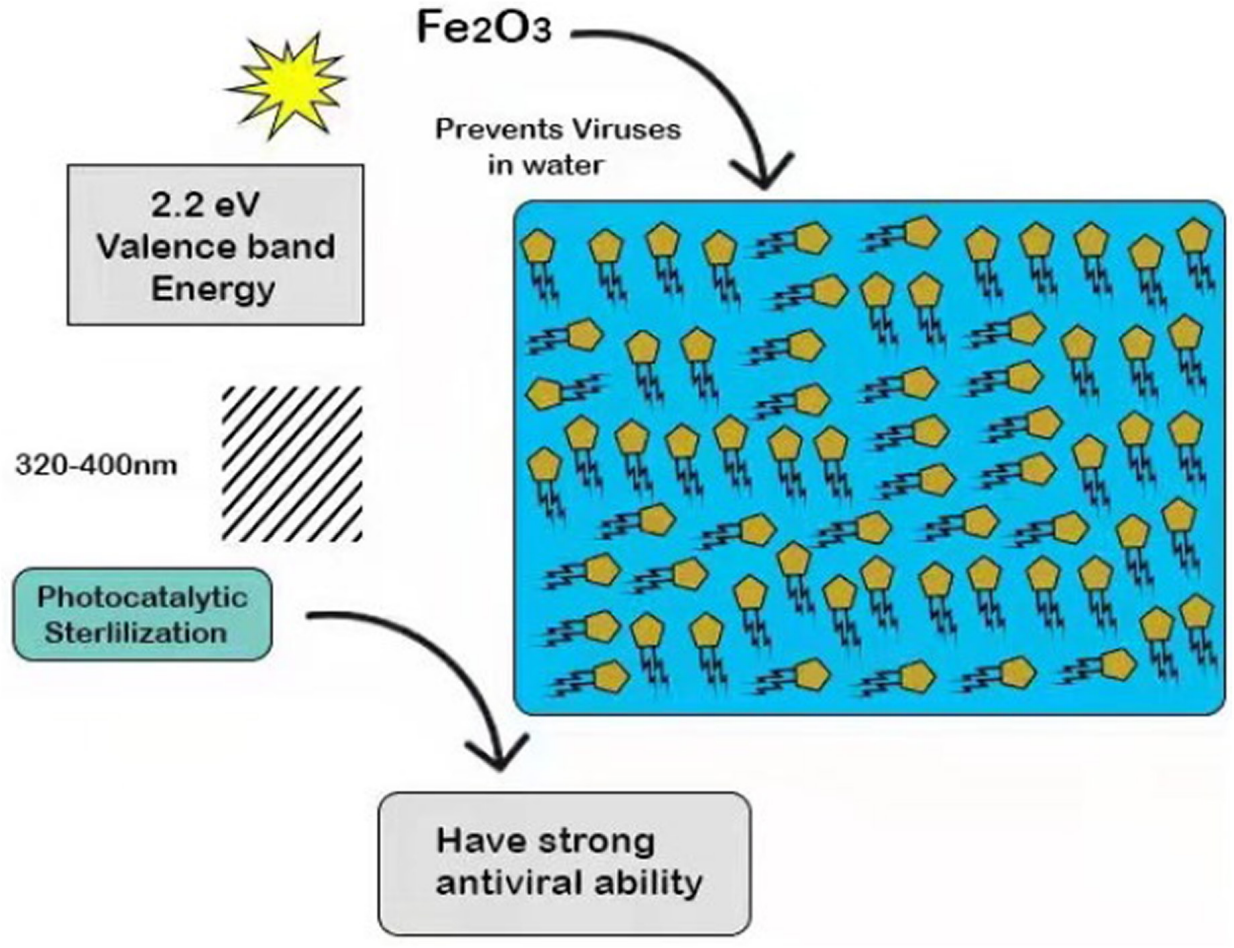
Antibacterial effect of nano‐Fe_2_O_3_ and its application.

Because the valence band of Fe_2_O_3_ is relatively narrow, it can be used as a sensor for TiO_2_ photocatalysis. When Fe_2_O_3_–TiO_2_ mixed films were irradiated under visible light, the valence band of Fe_2_O_3_ was excited from the valence band to the conduction band, leaving electron holes in the valence band (Ryan et al., 2002). In the implanted region of the TiO_2_–Fe_2_O_3_ covalent structure, excited electrons can be transmitted to each other to improve its activity. Figure 4 shows the details of nano‐Fe_2_O_3_ and its application for meat processing. According to the researcher (Nangmenyi et al., 2011), the antibacterial properties of TiO_2_ films decorated by Fe_2_O_3_ were higher than those of TiO_2_ films alone (Akhavan & Azimirad, 2009; Trapalis et al., 2003).

However, the antimicrobial activity of individual nano‐Fe_2_O_3_ is very low compared with that of titanium dioxide nanoparticles, which may be related to its low electron carrier capacity and the rapid recombination of photo‐activated substance pairs (Zhang et al., 2008). When Fe_2_O_3_ exists, H_2_O_2_ will exhibit a very strong photocatalytic effect, as reported earlier. Table 2 represents the antimicrobial effect of Fe_2_O_3_ by applying photocatalytic sterilization.

**TABLE 2:** Antimicrobial effect of Fe_2_O_3_ by applying photocatalytic sterilization.

Type	Light intensity	Wavelength	Targeted bacteria	Bacterial cell reduction	Food application	Action time	Antimicrobial effect	References
UV light illumination	—	380 nm	*Giardia lamblia*	∼106 CFU/mL	Packaging	120 min	Acts to kill pathogenic microbes and acts as antibacterial	Gong et al. (2019)
Visible light irradiation	320/cm	400 nm	*Staphylococcus aureus* and *Escherichia coli*	99.51% and 99.91%	Water disinfection	15 min	Reduces the microbes by exposing them to the food substances	Su et al. (2020)
Visible light	110 mW/cm^2^	450 nm	*E. coli*	2.5 log	Thin coating films	24 h	Causes bacterial inactivation	Akhavan and Azimirad (2009)
UV light	1650 W/m^2^	320–400 nm	*E. coli*	2 log	Packaging	2 h	Improves bactericidal activity	Karunakaran et al. (2013)
UV light	—	420 nm	*Bacillus subtilis*	—	Packaging	120 min	Decrease the formation of bacterial spores	Sánchez‐Salas et al. (2017)
Visible light	—	415–600 nm	*E. coli*	2 logs	Thin film packaging	210 min	Reduces the pathogenic bacteria	Jana et al. (2017)
Visible light	—	420 nm	*S. aureus*	—	Food Preservation	123 min	Acts as bacterial disinfection	Vignesh et al. (2019)
UV light	—	230–300 nm	*E. coli*	—	Food Packaging	1 h	Protects the shelf life of food commodity	Cui et al. (2013))
Visible light active	30 mW/cm^3^	420 nm	*E. coli*	1 log CFU	Packaging films	120 min	Acts as antimicrobial	Ouyang et al. (2016))
UV light	60 mW/cm^3^	415–600 nm	*E. coli*	3 log CFU	Food Preservation	1 h	Inhibit the bacterial spores' formation	Wang et al. (2016)

#### 5.2.3 Antibacterial effect of nano‐ZnO and its application

**FIGURE 5:**
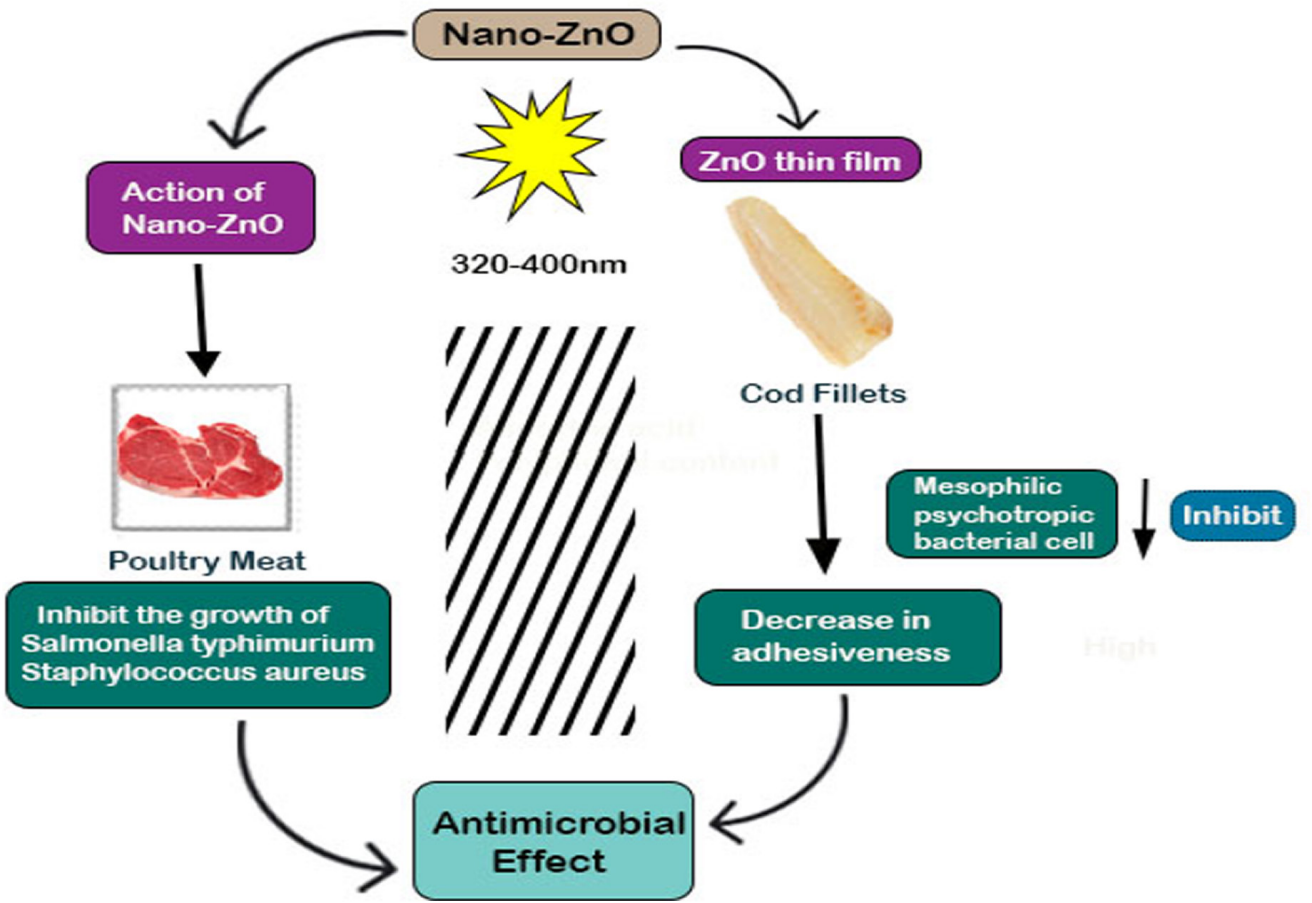
Antibacterial effect of nano‐ZnO and its application.

The synergistic effect of nano‐zinc oxide and nano‐polymer on microorganisms in food has been reported. The combination of nano‐ZnO and PVC can significantly inhibit the growth of *E*. *coli* and *Staphylococcus aureus* (Bandara et al., 2007). In a recent study, researchers (Li et al., 2009) applied nano‐zinc oxide film and found a significantly positive effect in terms of increased shelf life and stabilized quality parameters. Emamifar et al. (2010) studied the antimicrobial activity of low‐density polyethylene (LDPE) containing nano‐Ag and zinc oxide, which could significantly improve the quality and shelf life of the final product. In addition, it is also reported that the combination of allyl isothiocyanate with Nisin and nano‐ZnO in glass can effectively inhibit *Salmonella* (Emamifar et al., 2010). The antimicrobial action of nano‐ZnO on apple slices and poultry meat is illustrated below in Figure 5. Akbar and Anal (2014) reported the antimicrobial effect of ZnO nanoparticles against *S. aureus* and *Salmonella typhimurium* in poultry meat (Akbar & Anal, 2014). A study with the goal of integrating nano‐ZnO into packaging material to control Campylobacter in raw chicken meat was conducted. The author discovered that nano‐ZnO as active food packaging could control pathogens and prolong the shelf life of food without explicitly introducing antimicrobials to the food matrix (Moreau et al., 2008). A researcher developed novel polypyrrole–zinc oxide nanocomposite films based on modified bacterial cellulose (BC–PPy–ZnO) to store chicken thigh meat. The author found that the BC–PPy–ZnO film could increase the shelf life and stabilize the rheological properties of the chicken thigh by increasing antioxidant and antimicrobial activity as active packaging, helping us to estimate the storage period and storage temperature of the chicken thigh as smart packaging.

Similarly, various mechanisms have been suggested to explain the variations in the antibacterial activity of nano‐ZnO against Gram‐positive and Gram‐negative bacteria. More research is needed to establish the precise reason for these bacterial cells' vulnerability to nano‐ZnO. Furthermore, the antimicrobial activity of nano‐ZnO is influenced by surface area, particle size, and synergistic activity with other antimicrobial agents. Since particle size strongly affects nanoparticle functional behavior, nano‐ZnO with smaller particle size shows enhanced antibacterial activity against *S. aureus* and *E. coli* (Li et al., 2011). This could be due to the increased surface area‐to‐volume ratio of nano‐ZnO, which contributes to increased reactivity since H_2_O_2_ generation is highly dependent on surface area (Emamifar et al., 2010). In addition, on smaller nano‐ZnO particle surfaces, as the surface area decreases with particle size, a greater number of ROS are produced. Furthermore, theoretical experiments have shown that smaller particles can be more harmful to fungi and bacteria. This toxicity, however, can be due to a number of other variables, including particle morphology, surface chemistry, microorganism concentration, and light intensity (Jin & Gurtler, 2011). As a result, controlling for such external factors should be included in the studies in order to fully understand the effect of particle size on the toxicity of nano‐ZnO. Furthermore, the synergistic antimicrobial effects of nano‐ZnO, when combined with other antimicrobial agents, have attracted the interest of several researchers.

Antibacterial packaging is the future development direction, and nano‐antimicrobial packaging technology is one of the most widely used packaging types. Nano‐bacteriostasis packaging is a kind of packaging material that combines nanomaterials with polymer materials, or nanomaterials are added to the inner wall of packaging for product packaging. Nano‐packaging materials can inhibit the growth and reproduction of microorganisms on the food surface, prolong shelf life, and improve product safety. It will be one of the future development directions of food packaging. Therefore, with the increasing concern and requirement of consumers for food quality and safety, food packaging technology is developing in the direction of safety, convenience, rapidity, and no residue.

## 6 HIGH‐VOLTAGE ELECTRIC FIELD COLD PLASMA (HVEF‐CP) BACTERIOSTASIS AND PRESERVATION TECHNOLOGY

Plasma is a kind of material form consisting mainly of free electrons and charged ions that widely exist in the universe. It is called the fourth state of matter besides the solid, liquid, and gaseous states and is also called the “plasma state.” The plasma is composed of ions, electrons, and unionized neutral particles. The whole plasma is in a neutral material state.

**FIGURE 6:**
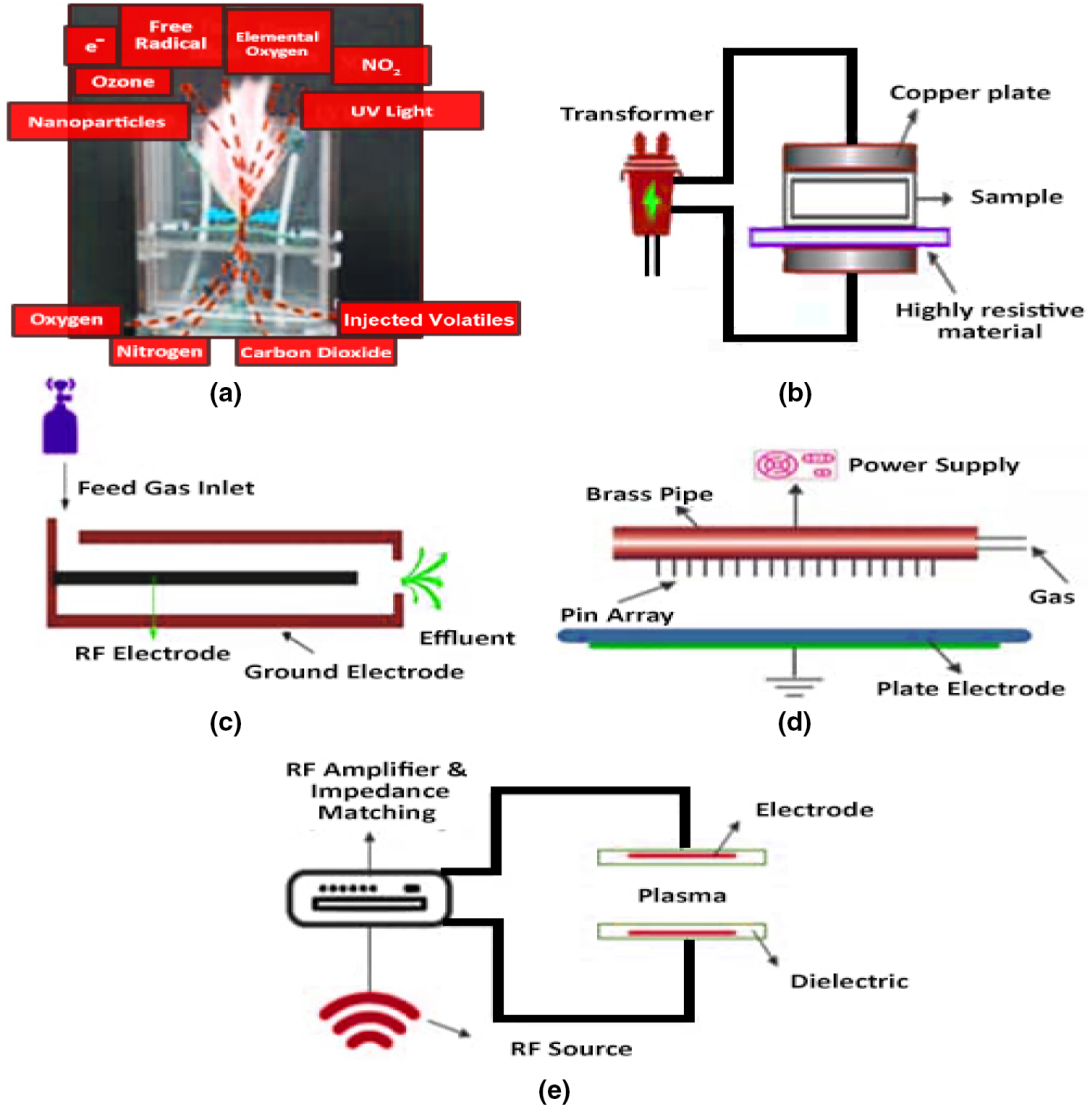
(a) plasma generation–ionization processing, (b) resistive barrier discharge (RBD) (c) the atmospheric pressure plasma jet (APPJ), (d) corona discharge system, (e) configuration of the DBD‐based diffuse glow discharge atmospheric pressure.

The difference between plasma and ordinary gas is that ordinary gas is made up of molecules, and only intermolecular forces exist; plasma is a neutral gas containing ionized substances, and the plasma generation composition and different sources of plasma generation have been shown in Figure 6a–e (Umair et al., 2021). The plasma formation process can be simply described as follows: a neutral gas is ionized by enough electric energy. When the energy exceeds the charge force between atoms of gaseous molecules, free electrons will be stimulated, and electrons will collide with the surrounding atoms and molecules to produce more active excited atoms, ions, and electrons. During collisions, some of the energy is converted into the form of light energy. The temperature of plasma does not increase significantly at low ionization, which is close to room temperature, so it is called cold source plasma (Moreau et al., 2008). Table 3 shows the bactericidal action of high‐voltage electric field plasma sterilization technology.

**TABLE 3:** Bactericidal action of high‐voltage electric field plasma sterilization technology.

Plasma generating medium	Plasma mixture	Targeted bacteria	Log CFU/mL	Exposure duration	Result	References
Atmospheric cold plasma (air)	Oxygen source	*Escherichia coli* and *Staphylococcus aureus*	3.6‐ and 2.3‐log (2 log)	3 min	Cell envelop, causes shrinkage of cell	Umair et al., (2022)
Low‐pressure oxygen plasma	Oxygen source	*Salmonella typhimurium*	6.3‐ and 3.4‐log (3 log)	600 s	Acts as a sanitizer to produce quality fresh vegetables	Zhang et al. (2013)
Helium plasma treatment	Helium and argon	*Bacillus subtilis* and *Yersinia enterocolitica*	2.09–0.98 log (2 log)	2 min	Inactivates various meat microbiota	Ulbin‐Figlewicz et al. (2015)
High‐voltage atmospheric cold plasma	Reactive oxygen species, ozone	*Listeria monocytogenes*	1.5 log	60 s	Prevents the bacterial cells by inhibiting their reaction	Han et al. (2016)
Dielectric barrier discharge plasma	Helium and Oxygen	*E. coli* and *L. monocytogenes*	1.5 log	5 or 10 min	Reduces the impact of pathogenic bacteria	Kim et al. (2013)
Radiofrequency atmospheric pressure plasma	Oxygen	*S. aureus*	3–4 log	2 min	Inactivates the bacterial growth on the meat surface	Kim et al. (2014)
Atmospheric pressure fluidized bed plasma	Air and Nitrogen	*Aspergillus* spp.	3 log	3 min	Reduces the bacterial growth on grains	Dasan et al. (2016)
Radiofrequency atmospheric pressure	Argon Plasma	*Pseudomonas aeruginosa*	3 log	13 min	Retards the bacterial growth	Van Gils et al. (2013))
Radiofrequency plasma	Nitrogen and Oxygen mixture	*S. aureus*	6 log	5 min	Controls the adverse consequences imposed by bacterial proliferation	Sureshkumar et al. (2010)
High‐voltage atmospheric cold plasma	Oxygen and nitrogen	*L. monocytogenes*	2 log	30 s	Retards the bacterial growth and kills the pathogenic microorganisms	Lu et al. (2014)
Low‐temperature atmospheric plasma	Argon	*Micrococcus luteus*	3 log	2–3 min	Damages the structure of bacterial cell	Yu et al. (2007)
Atmospheric pressure plasma	Helium, Nitrogen, and Oxygen	*L. monocytogenes*	2 log	2 min	Extends the shelf life of food products by inactivating bacteria	Lee et al. (2011)
Cold atmospheric pressure plasma	—	*S. enterica, B. subtilis* spores	2.8 log	30 min	Improves the quality of spices	Hertwig et al. (2015)
Nonatmospheric pressure plasma	Plasma‐treated water and air	*E. coli*	1.6 log	1–2 min	Acts as biological decontamination	Schnabel et al. (2019)
Plasma jet	Plasma‐treated active solution	*E. coli*	3–5 log	5–45 s	Improves the quality and color characteristics of spinach and acts as antimicrobial	Feizollahi et al. (2021)

The plasma can be produced in a wide range of atmospheric pressure and temperature as well as in other different resource conditions, such as microwave, pulse, AC, and DC. In recent years, nonthermal source plasma and atmospheric (atmospheric) plasma have been widely discussed because they can be used in a variety of technologies, such as polymer surface modification and biological and chemical decontamination (Moreau et al., 2008). Currently, plasma is a mixture of neutral and ionized particles, metastable groups, and free radicals. Depending on the type or types of plasma, its energy content, and the substance produced or modified, the plasma effect is affected by its composition (Moreau et al., 2008). Because it is difficult to apply plasma in a fully controlled range, the efficiency of plasma generation can be changed according to the specific plasma state and the change in plasma/matter interaction.

### 6.1 Active substances in high‐voltage cold plasma and influencing factors of bactericidal action

#### 6.1.1 Ozone

Ozone, one of the many active antimicrobial substances produced in the oxygen‐containing gas plasma system, has a relatively long life to enable it to be quantitatively determined over time. It is reported that more than 75 active substances and nearly 500 reactions (Wang et al., 2002) can be generated in the air plasma. These reactions are produced in four different time ranges: nanoseconds, microseconds, milliseconds, and seconds. It is difficult to determine all the active groups. Further research is needed. It is worth noting that ozone is generally recognized as safe by the Food and Drug Administration (FDA) as a direct food additive (FDA, 2001; Gordillo‐Vázquez, 2008). The DBD system is considered to be one of the most effective ways to produce ozone (Rice & Graham, 2001). The electrons generated by DBD in the ionized air inside the packaging break the molecular bonds of oxygen into separate oxygen atoms, which then combine with oxygen to produce ozone.

Nonthermal processing is a way to enhance food safety without reducing quality or the required quality (Umair et al., 2020). The use of gaseous ozone to kill pathogens is a feasible way to maintain food quality and enhance food safety. According to Guzel‐Seydim et al. (2004) reviews of ozone in food, the food industry has begun to find a better way to enhance food safety. Ozone is an effective bactericidal gas and does not produce much residue. Ozone is produced by using a specific voltage, frequency, and geometry between the two electrodes. A better method is to use double‐layer dielectric barrier discharge to generate ozone. Reactive oxygen species are generated, which react with each other and with oxygen atoms to produce ozone. These reactive oxygen groups form in air or oxygen, including ozone, singlet oxygen, superoxide anions, peroxide particles, or hydroxyl radicals (Hakeem et al., 2020). Most of the particles exist for a very short time (milliseconds) and cannot work well, but the existence time of ozone is relatively long; depending on different treatment conditions, it can exist for several minutes to several days (Pirsa & Shamusi, 2019). Another scientist believes that ozone is more effective than chlorine‐containing disinfectant water in terms of low concentration and treatment times. Singh et al. (2002) treated with ozone gas for 15 min could kill *E. coli* O_157_:H_7_ of 1.79 log CFU/g on meat surfaces, which proved that gas state treatment was more effective than liquid state treatment. Bialka and Demirci (2007) showed that gaseous ozone had similar effects on O_157_:H_7_ and *Salmonella* after treatment. Akbas and Ozdemir (2008) believe that ozone treatment for 360 min can reduce *E. coli* and *Bacillus cereus* by 3.5 logs. Meanwhile, *Bacillus cereus* can be reduced by 2 logs on the drying film.

#### 6.1.2 Role of ultraviolet

The bactericidal effect of ultraviolet light is mainly realized by the absorption of ultraviolet photons by proteins in microorganisms, resulting in their molecular denaturation and inactivation. By comparing and analyzing the kinetics of ultraviolet irradiation in low‐pressure xenon lamps and atmospheric cold source plasma, they found that when the bacteria were exposed to an atmospheric pressure plasma beam (APPJ), glass was used to isolate the plasma, and no significant decrease in the number of bacteria was found (Bialka & Demirci, 2007). The bactericidal effect of ultraviolet itself mainly needs certain conditions: the bactericidal effect of ultraviolet light on bacteria needs an appropriate wavelength (280–220 nm), and the light intensity should be high enough. If the ultraviolet light produced during plasma treatment cannot meet the above conditions simultaneously, it will not have a bactericidal effect. When the distance of plasma treatment is large, the short‐wavelength ultraviolet light produced by plasma treatment, its transmission distance, and its penetration depth are not enough to produce a lethal killing effect on bacteria. Roth et al. (2010) found that a certain amount of Ultraviolet‐C light can be produced in plasma, which is one of the effective active ingredients in plasma sterilization.

#### 6.1.3 Mode of action of other active substances

Many active substances are produced during plasma treatment, among which oxygen‐containing active substances are proven to have the strongest effect on microorganisms. Although ozone has existed for a long time and plays a major role, other short‐term active substances will also impact microorganisms, especially those with high oxidation. Oxygen‐containing active substances are easily oxidized and denatured with proteins and nucleic acids in microorganisms, resulting in the death of all kinds of microorganisms (Umair et al., 2021).

**FIGURE 7:**
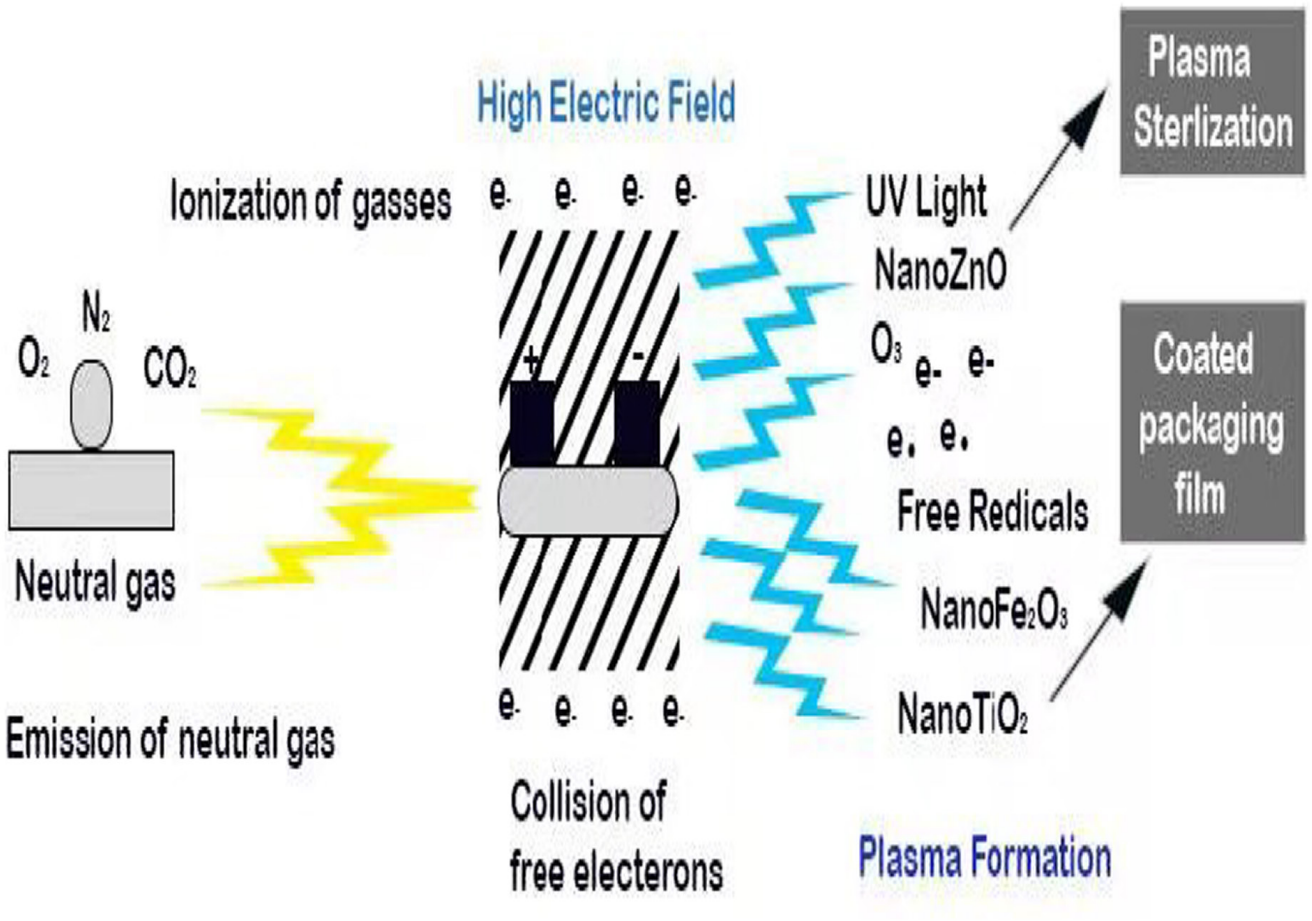
Mode of action of reactive substances in plasma in high voltage cold plasma.

At the beginning of treatment or the end of treatment, other active antimicrobial groups may participate in or enhance the sterilization of ozone. Nitrogen‐containing active groups produced by nitrogen may enhance bactericidal action. Nitrogen‐containing active groups may react with components in suspension to produce nitric acid or nitrite, decreasing pH value and thereby enhancing plasma sterilization. Oehmigen et al. (2010) found that nitrogen‐containing substances could reduce pH and that pH significantly affected the number of visible bacteria. In addition, the hydrogen peroxide produced during the treatment will enhance the bactericidal effect of the solution. Hydrogen and oxygen groups can attack the cell wall, destroy it, and affect the normal growth of bacteria. Perni et al. (2007) used photoexcitation spectroscopy to study bacteriostatic dynamics. The hydroxyl group, singlet oxygen group, and nitrogenous group play a less important role than the ozone group in plasma (Figure 7).

In addition, plasma contains a large number of electrons and charged groups, as well as charged ions, which may also affect microorganisms in the process of plasma sterilization. Although these active substances do not play a major role in plasma sterilization, like ultraviolet light, they still impact the sterilization effect (Ikawa et al., 2010). Electrons and charged ions have high energy during plasma generation, which can produce a breakdown etching effect on the cell wall of bacteria. Mendis et al. (2000) and others believe that these charged substances may play a significant role in the rupture process of the cell membrane and cell wall because these charged substances can accumulate aggressive force in the outer membrane and ultimately destroy the tensile strength of the outer membrane, leading to cell rupture (Oehmigen et al., 2010).

### 6.2 Bactericidal effect of plasma

#### 6.2.1 Bactericidal effect of air source plasma

As a complex mixture, plasma has many factors affecting its production, although the equipment is simple. Plasma formation mainly depends on the equipment, operating conditions, and gas composition, which determine the efficiency of plasma production. Among them, the production equipment mainly refers to the shape of the reactor used for plasma generation, and the shape and size of the electrodes will affect the production of plasma. The operating conditions mainly refer to the gas pressure, velocity, excitation voltage, and frequency. Operating conditions are controllable, and plasma formation can be controlled by changing one or more conditions. The gas composition determines the main components of plasma. According to Oehmigen et al. (2010), an oxygen source produces more effective plasma than a pure nonoxygen source (argon and helium).

How the treated substrate is exposed to the plasma has an important influence on the overall effect of the plasma. The direct or indirect exposure of the treated substrate to the plasma, or the distance between the treated substrate and the plasma‐producing region (Bialka & Demirci, 2007; Perni et al., 2007), is considered to be an important factor. If the substrate is exposed far away, the amount of heat transferred to the sample surface in the plasma decreases; the effect of charged substances on the sample decreases significantly because they recombine or react before reaching the sample surface; and most short‐lived, highly active substances disappear before reaching the sample surface. Because the composition of plasma is highly active and self‐destructive (X. Lu et al., 2008) due to its short half‐life, reducing the time of the sample reaching the surface of the sample becomes the main factor affecting the sterilization effect of plasma.

In addition, the water content of the sample and the moisture content of the package affect the production of the plasma. When the relative humidity in the gas reaches a certain level, the plasma effect is enhanced (Mendis et al., 2000). The wet state is conducive to the transfer of free electrons between different groups. Materials produced by plasma may affect their formation.

#### 6.2.2 Bactericidal action of nonoxygen source plasma

When the gas produced by plasma is not air or the oxygen content is low, the ozone production in plasma obviously decreases. In addition to ozone, the active bactericidal substances produced during plasma treatment have a short half‐life, a short existence time, and a relatively short duration of killing microorganisms. In this case, factors such as the presence of bacteria (suspension liquid or dry bacterial film) and the ultraviolet light produced may increase the effect on the overall germicidal efficacy of plasma. Therefore, the use of nonoxygen plasma makes it easy to study the bactericidal effect of ozone (Niemira & Sites, 2008).

Weng et al. (2009) dripped 107 CFU/mL bacterial suspensions onto PDMS membranes and treated them with plasma after drying. The changes in active ions, ultraviolet light intensity, reaction temperature, and bacterial colonies were studied. It was found that the effect of adding 0.5% oxygen for 30 s on *E. coli* was the best in DBD equipment. The increase in the number of excited oxygen ions did not enhance the sterilization effect of the argon plasma beam. On the contrary, prolonging the treatment time of bacteria in plasma is more important for enhancing the bactericidal effect. The ratio of oxygen to argon produced by atmospheric plasma treatment changes. Adding oxygen to argon can increase the number of active groups. The bactericidal effect of the current DBD plasma system is likely to depend on the ratio of active oxygen to argon. A small amount of oxygen acts as the reaction component in the plasma system, but the main factor for the sterilization effect of the plasma is the treatment time (Fridman et al., 2007; Ragni et al., 2010; Tanino et al., 2007).

### 6.3 Application of plasma in the food industry

Compared with heat source sterilization, cold source plasma is especially suitable for heat‐sensitive food sterilization. The sterilization of plasma in the food industry will have an important impact on the safety of packaged food and the shelf‐life extension. Plasmas can be applied to sterilize food surfaces (such as meat, poultry, and fish) (Eto et al., 2008; Fridman et al., 2007; Heise et al., 2004; Leipold et al., 2010).

Studies on the bactericidal effect of plasma have been reported. Critzer et al. (2007) used plasma to treat pathogenic bacteria *E. coli* O157:H7, *Salmonella* sp., and *Listeria monocytogenes* on the surface of chicken meat. The number of colonies decreased significantly in all samples, and the reduction varied with the strains. Perni et al. (2008) have reported the effect of cold plasma generation by DC voltage and inoculated them with *E. coli*, *Saccharomyces cerevisiae*, *Pantoea agglomerans*, and *Gluconacetobacter liquefaciens*. The results showed that *S. cerevisiae* had the strongest resistance to the treatment conditions. The increasing voltage could produce a more effective plasma effect and enhance the effect on bacteria (Eto et al., 2008). Similarly, Niemira and Sites used a sliding‐arc medium to produce plasma. After treating chicken inoculated with *Salmonella* and *E. coli* O_157_:H_7_ on the surface, the number of colonies decreased significantly. *Salmonella* and *E. coli* decreased by 2.9–3.7 and 3.4–3.6 log CFU/mL, respectively. They believed that the highest air flow rate, such as 4.4–3.6 log CFU/mL, was the plasma source, and 0 L/min showed the best effect (Schwabedissen et al., 2007).

The study of plasma sterilization on eggshell surfaces has been reported in the literature. Ragni et al. (2010) studied the bactericidal effect of resistive dielectric plasma (RBD) on the meat surface. It was found that the reduction of *Salmonella enteritidis* on meat surfaces could reach 2.2–2.5 log CFU/eggshell after 60–90 min of treatment at 35% relative humidity (RH). They also found that when the relative humidity increased to 65%, the reduction of bacteria increased to 3.8–4.5 log CFU/g of meat after 90 min of treatment (Stoffels et al., 2008).

There are also reports on the bactericidal effect of plasma on meat products. Laroussi and Leipold (2004) studied the bactericidal effect of atmospheric pressure cold source plasma on the surface of sliced bacon. They inoculated the surface of sliced bacon with *Listeria monocytogenes* (KCTC 3), *Escherichia coli* (KCTC 1682), and *S. typhimurium* (Laroussi et al., 2003). To make them sterile, the samples were treated with different input voltages of 75, 100, or 125 W for 60 or 90 s. A mixture of helium, helium, and oxygen produces gas for plasma. The results showed that the number of pathogenic bacteria decreased by 1–2 logs after helium source plasma treatment, while it decreased by 2–3 logs after helium or oxygen source plasma treatment. At the same time, it was found that the microstructures of bacon did not change significantly after plasma treatment, except for the increase in the L* value (Weng et al., 2009).

Thus, food sensitive to heat sources is often prone to spoilage due to incomplete sterilization, which shortens the shelf life and is not conducive to long‐distance transportation. Cold‐source plasma technology can produce high‐efficiency bactericidal substances under low voltage, which can effectively sterilize the surface of products. At present, there are few studies on the bactericidal effect of food packaging (Yu et al., 2006). Fresh meat is not suitable for heat‐source sterilization. It is significant for meat and meat products and the whole food industry to develop a cold sterilization method for heat‐sensitive food. In this study, the photocatalytic sterilization and cold source plasma sterilization of nanomaterials were studied, and the synergistic effect of the two materials was preliminarily explored, which provided basic theoretical support for the development of photocatalytic antimicrobial packaging material and methods and technical support for the exploration of cold sterilization technology, fresh meat, and heat. This provides a reference for fresh food packaging technology for sensitive foods (Critzer et al., 2007).

## 7 CONCLUSION

At present, the packaging technology and sterilization methods of heat‐sensitive foods, such as fresh meat, are not thoroughly sterilized. Despite low‐temperature treatment during storage and transportation, surface microorganisms can still proliferate in large numbers, causing spoilage and short shelf life. Besides, the temperature change during the thermal sterilization process impacts the sensory quality, physical and chemical properties, and nutritional components of the product, which is not conducive to the maintenance of the original quality. To sterilize fresh meat more effectively, extend its shelf life, and maintain its original quality (original taste), it has become the requirement and trend of the food industry to develop efficient cold sterilization methods and antimicrobial packaging technology. Ultraviolet light penetration ability is weak; normal use of packaging boxes or food packaging materials can block the penetration of ultraviolet light; that is, most food packaging currently in use cannot penetrate ultraviolet light. Because the packaging cannot penetrate ultraviolet light, the use of nanoparticles with photocatalytic and bacteriostatic activity as antibacterial packaging is limited. So how to contain ultraviolet light in the packaging is an important factor in using nano‐photocatalytic antibacterial packaging and extending product shelf life effectively. Therefore, as a new method of cold sterilization, plasma sterilization technology may be used to sterilize fresh meat and heat‐sensitive food. In addition, plasma contains many active substances and can also produce ultraviolet light. Ultraviolet light may stimulate the activity of metal nanomaterials. If nanomaterials are combined with plasma technology to effectively stimulate the activity of nanomaterials, it will promote the inhibition of microorganisms on the surface of packaged meat products.

## AUTHOR CONTRIBUTIONS

**Muhammad Umair:** Conceptualization (equal); data curation (equal); writing – original draft (equal). **Tayyaba Sultana:** Conceptualization (equal); resources (equal); software (equal). **Song Xun:** Conceptualization (equal); funding acquisition (equal); writing – original draft (equal). **Saqib Jabbar:** Resources (equal); software (equal); writing – review and editing (equal). **Muhammad Shahid Riaz Rajoka:** Conceptualization (equal); resources (equal); software (equal); writing – review and editing (equal). **Amgad Albahi:** Methodology (equal); resources (equal); software (equal); writing – review and editing (equal). **Muhammad Abid:** Conceptualization (equal); investigation (equal); methodology (equal); writing – original draft (equal). **Muhammad Modassar Ali Nawaz Ranjha:** Data curation (equal); software (equal); writing – original draft (equal). **Hesham R. El‐Seedi:** Project administration (equal); resources (equal); writing – review and editing (equal). **Fengwei Xie:** Project administration (equal); supervision (equal); writing – review and editing (equal). **Kasif ur Rehman Khan:** Conceptualization (equal); methodology (equal); software (equal); writing – review and editing (equal). **Liqing Zhao:** Funding acquisition (equal); project administration (equal); visualization (equal); writing – review and editing (equal). **He Zhendan:** Conceptualization (equal); funding acquisition (equal); project administration (equal); resources (equal); supervision (equal).

## CONFLICT OF INTEREST STATEMENT

The authors declare that they have no conflict of interest.

## Data Availability

The data that support the findings of this study are available on request from the corresponding author.
